# Recognition of a New Cr(VI)-Reducing Strain and Study of the Potential Capacity for Reduction of Cr(VI) of the Strain

**DOI:** 10.1155/2019/5135017

**Published:** 2019-02-10

**Authors:** Chunyong Wang, Yanshan Cui

**Affiliations:** ^1^College of Resources and Environment, University of Chinese Academy of Sciences, Beijing 101408, China; ^2^Research Center for Eco-Environmental Sciences, Chinese Academy of Sciences, Beijing 100085, China

## Abstract

The biotransformation of hexavalent chromium [Cr(VI)] via Cr(VI)-reducing microorganisms is considered an ecofriendly approach to detoxify Cr(VI). A new Cr(VI)-reducing bacterium named* Microbacterium* sp. QH-2 was isolated in this study. Scanning electron microscopy (SEM) images showed protrusions on the bacterial surface of strain QH-2 after an 18 h incubation in media under 10 mM Cr(VI) treatment. Results of the experiments on the capacity of reducing Cr(VI) indicated that strain QH-2 could reduce 100% Cr(VI) less than 48-96 h. When media with 4 mM Cr(VI) were incubated, the fastest reduction rate of strain QH-2 could come up to 2.17 mg/L Cr(VI) h^−1^. Furthermore, strain QH-2 could reduce Cr(VI) over the pH between 7 and 10. The optimum pH to reduce Cr(VI) by strain QH-2 was 9. Strain QH-2 also exhibited a relatively high tolerance even to 20 mM Cr(VI). These results declared that strain QH-2 had the potential to detoxify Cr(VI) in the Cr(VI)-contaminated soil or effluent.

## 1. Introduction

In recent years, because of its use in pigments, ceramics, leather tanning, metal corrosion inhibition, and refractory materials, environmental pollution caused by chromium (Cr) in soil and sediments, etc. is widespread [12, 13]. According to statistics, China produces the largest amount of Cr slag, reaching as much as 450,000 tons per year [14]. If Cr slag is not properly treated or is directly released into the environment, it may pollute the environment. Soil is often used as sites for the depositing of Cr slag, so this soil has a high concentration of dissolvable hexavalent chromium [Cr(VI)] [15]. It has a toxic effect on agricultural plants due to the fact that it could be absorbed by the roots. It was reported that plants suffered high toxicity when the concentration of Cr(VI) reached 100 mg/kg in soil [16]. Furthermore, Cr(VI) is weakly adsorbed by soil particles and can easily enter surface water or groundwater. Therefore, it is urgent to take measures to detoxify Cr(VI) in the environment.

Except for Cr(VI), trivalent chromium [Cr(III)] is also a major present form in the environment. Cr(III) has a low solubility. In addition, it can act as a nutrient for organisms and microorganisms [17, 18]. By contrast, Cr(VI) is soluble, mutagenic, and teratogenic. The conversion of Cr(VI) to Cr(III) is related to microflora, organic compounds, pH, etc. in the environment [16]. Promoting the conversion of Cr(VI) to Cr(III) is considered to be a workable way to reduce the toxicity of Cr(VI). Especially in the soil environment, Cr(III) can be immobilized by soil due to the adsorption of Cr(III) by soil [19]. This method not only reduces the toxicity of Cr(VI) but also reduces the free Cr(VI) and prevents it from entering and contaminating the groundwater.

At present, the chemical reduction is the widely used method to reduce the toxicity of Cr(VI). Common chemical reductants include ferrous sulfate [20] and elemental sulfur (S^0^) [21]. Additionally, selective adsorbing materials such as treated sawdust [22], biochar [23], and modified activated carbon [24] can also be applied to remove Cr(VI). However, those methods could lead to the high costs associated with chemical consumption and low efficiencies [19]. Therefore, finding a new way to reduce Cr(VI) is very urgent.

The biotransformation of Cr(VI) is considered to be capable of being performed by Cr(VI)-reducing bacteria and fungi, which can be isolated under aerobic and anaerobic conditions. Various Cr(VI)-reducing bacteria such as* Cellulosimicrobium* sp. [25],* Stenotrophomonas maltophilia* [26], and* Alishewanella* sp. WH16-1 [27] have been isolated from the tannery effluent and the mining soil. Meanwhile, some fungi such as* Pisolithus* sp1 [28] and* Penicillium oxalicum* SL2 [29] can reduce Cr(VI). These bacteria tolerate Cr(VI) while reducing Cr(VI) in the Cr(VI) contaminated environment. In short, the biotransformation of Cr(VI) via Cr(VI)-reducing bacteria and fungi has provided an ecofriendly and low-cost approach to resolve the problem of Cr(VI) pollution [12].

In present study, we aim to (i) isolate a new Cr(VI)-reducing bacteria from Cr(VI)-contaminated alkali soil under aerobic conditions, (ii) study the morphological changes of the bacterial strain under Cr(VI) and the phylogenetic tree analysis, and (iii) evaluate both the capacity to reduce and resist Cr(VI) by the bacterial strain.

## 2. Materials and Methods

### 2.1. Enrichment and Isolation of Bacterial Strain

Soil samples were collected from the alkali soil contaminated with Cr(VI) because of the stacking of Cr slag in Qinghai Province, China. The pH of the soil (pH 9.85) was measured by using a soil to water ratio of 1:2.5. Total Cr in soil was 2267.1 mg/kg. To enrich Cr(VI)-reducing bacterial strains, 100 g of soil was incubated with 5 mM K_2_CrO_4_ under aerobic conditions at 30°C for 1 week. During the incubation period, sterile water was infused to keep the soil hydrated. After the incubation, 10 g of soil was added to 100 mL of 0.85% NaCl solution then shaken at 30°C for 2 h. After the shaking process, the soil extract was taken. The soil extract was serially diluted, then spread on Luria-Bertani (LB) plates with K_2_CrO_4_ [34]. Then, LB plates were cultured at 30°C. The pure strains were obtained after several rounds of isolating single colonies.

### 2.2. Recognition of the Bacterial Strain

DNA was extracted via the FastDNA Spin Kit based on the description then dissolved in Tris-EDTA buffer. Then the mixture was stored at -20°C before PCR amplification. The 16S rRNA genes were amplified with 27F and 1492R [37]. Conditions of PCR amplification were as follows: 5 min at 95°C; then 30 cycles of 30 s at 95°C, 30 s at 55°C, and 90 s at 72°C; then 10 min at 72°C and a hold at 4°C. After the amplification, the amplified products were checked via using 1.0% agarose gel electrophoresis. Sequencing was achieved via Beijing Majorbio Sanger Bio-Pharm Technology Co., Ltd., Beijing, China.

### 2.3. Scanning Electronic Microscopy (SEM)

The bacterial strain was inoculated in the fresh LB media with 10 mM Cr(VI) for the study of the influence of Cr(VI) to the cell morphology via SEM. The media were incubated for 18 h (30°C, 160 rpm). The bacterial cells were centrifuged (8000 rpm, 10 min). After the centrifugation, cells were taken then washed with phosphate buffer saline (PBS) for three times (wash every fifteen minutes). After the washing process, cells were fixed with 2.5% glutaraldehyde then stored at 4°C. Before the SEM, the glutaraldehyde should be removed from the cells. Hence, the cells were washed with ultrapure water for three times. Then, the bacterial cells were dehydrated with 50%, 70%, 85%, 95%, and 100% (V/V) ethanol and dried. Finally, the cell samples were coated with gold and the morphology of the cell samples was examined with SEM (Hitachi SU8010, Japan).

### 2.4. The Capacity to Reduce Cr(VI) by the Bacterial Strains

In order to study the Cr(VI)-reducing capacity of the bacterial strains, 10 mL of logarithmic phase bacterial cultures grown in different initial concentrations of Cr(VI) (1 mM - 4 mM) was injected into the fresh LB media (100 mL, pH 9.0). Fresh LB media supplemented with the same initial Cr(VI) concentrations, but not inoculated with the bacterial cultures, served as controls. Culture media were incubated (30°C, 160 rpm). Samples of culture media were aseptically withdrawn at different time point then centrifuged (6000 rpm, 10 min) to detect the optical density at 600 nm (OD_600_) via the spectrophotometer (UV-4802H, UNIC, China). The culture supernatant was taken and then passed through a 0.22 mm filter for detecting the amount of Cr(VI) remaining in media via the 1,5-diphenylcarbazide (DPC) method at 540 nm (GB/T7467-1987, China). In brief, sulfuric acid (H_2_SO_4_) and phosphoric acid (H_3_PO_4_) were diluted with ultrapure water (1:1, V/V), respectively. Then 0.2 g of DPC was dissolved in 50 mL acetone and diluted with ultrapure water to 100 mL. This solution was used as the chromogenic reagent. The 0.2829 of K_2_Cr_2_O_7_ (dried at 110°C for 2 h) was dissolved with ultrapure water in a 1000 mL volumetric flask which was used as the Cr (VI) stock solution. The Cr (VI) standard solution was by appropriate dilution of the Cr (VI) stock solution mixed with 0.5 mL dilute H_2_SO_4_, 0.5 mL dilute H_3_PO_4_, and 2 mL chromogenic reagent. The culture supernatant of sample was centrifuged then mixed with the same solutions above in a 50 mL colorimetric tube. The mixture was measured at OD_540_ and the ultrapure water was used as a reference. Remaining Cr(VI) was considered to reflect the capacity for Cr(VI) reduction and calculated as

(1) $$\begin{array}{c} \text{Remaining Cr(VI)}\left(\;\%\;\right)=\left(\;\frac{A}{A_{0}}\;\right)\times 100\% \end{array}$$

where A is the remaining Cr(VI) in the culture supernatant and A_0_ is the Cr(VI) in controls.

### 2.5. Influence of Different pH to Reduce Cr(VI) by Bacterial Strains

Batch tests were performed to discuss the influence of different pH to reduce Cr(VI). Fresh LB media were adjusted to targeted pH (6, 7, 8, 9, and 10) using HCl or NaOH. Then, the media were inoculated with logarithmic phase bacterial cultures and supplemented with 5 mM Cr(VI). Culture media were kept in a rotatory shaker (30°C, 160 rpm). Noninoculated controls adjusted to pH and supplemented with Cr(VI) were incubated in the same conditions. Samples of culture media were collected at different intervals to detect the OD_540_ then centrifuged (6000 rpm, 10 min) to detect the remaining Cr(VI).

### 2.6. Evaluation of the Resistance of Bacterial Strains to Cr(VI)

For the evaluation of resistance of bacterial strains to Cr(VI), logarithmic phase bacterial cultures and different concentrations of Cr(VI) (6 mM, 8 mM, 10 mM, 15 mM, and 20 mM) were added to the fresh LB media (pH 9.0). Culture media were kept in a rotatory shaker with the same conditions as described above. The OD_600_ was monitored at different intervals. Inoculated media without Cr(VI) served as controls.

### 2.7. Phylogenetic Tree Analysis and GenBank Accession Number

The nucleotide sequence of strain QH-2 was submitted to GenBank (MH319855.1). Then the nucleotide sequences of strain QH-2, some* Microbacterium *species in the BLAST programme, and some reported strains that can reduce Cr(VI) were selected to establish the phylogenetic tree via MEGA 4.0 software.

### 2.8. Statistical Analysis

Experiments of this study had three replicates in order to calculate the standard deviations.

## 3. Results

### 3.1. Recognition and Morphology of Cr(VI)-Reducing Bacteria

**Figure 1:**
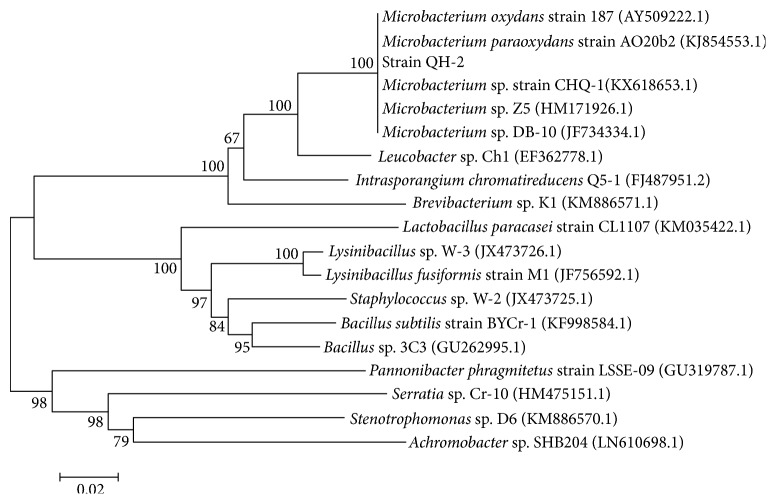
The neighbour-joining phylogenetic tree reflected the phylogenetic relationship of strain QH-2 with some* Microbacterium *species in BLAST and other Cr(VI) reduction strains reported by Zhu et al. [1], Liu et al. [2], Ge et al. [3], Huang et al. [4], He et al. [5], Raja et al. [6], Zheng et al. [7], Hong et al. [8], Xu et al. [9], Zhang et al. [10], and Rao et al. [11].

A number of bacterial strains were isolated through aerobic cultivation from several single colonies in this study, and the strain QH-2 which had a strong capacity for reduction Cr(VI) was selected. Based on BLAST analysis, strain QH-2 belonged to the* Microbacterium* genus. The neighbour-joining phylogenetic tree of strain QH-2 is depicted in Figure 1. Results showed that the nucleotide sequence of strain QH-2 revealed high similarities to that of* Microbacterium* sp. strain CHQ-1 (KX618653.1) and* Microbacterium *sp. Z5 (HM171926.1), etc. Consequently, strain QH-2 could be characterized and named as* Microbacterium* sp. QH-2 (MH319855.1).

**Figure 2:**
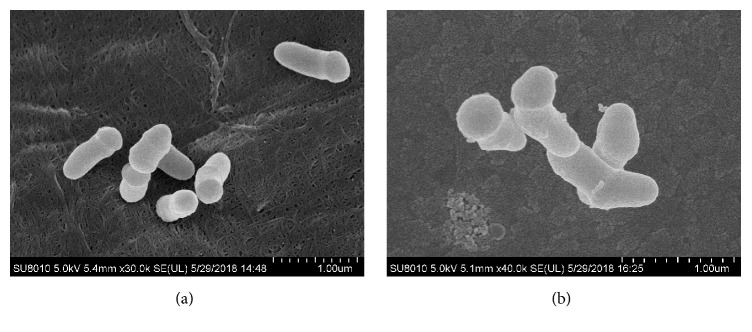
SEM images of strain QH-2 separated from the LB media. (a) Strain QH-2 from the media without Cr(VI) after 18 h of incubation. (b) Strain QH-2 from the media amended with 10 mM Cr(VI) after 18 h of incubation.

The SEM images of strain QH-2 from the media supplemented with 10 mM Cr(VI) and without Cr(VI) are exhibited (Figure 2). Strain QH-2 cells were rods and had a smooth bacterial surface (Figure 2(a)). However, protrusions were observed on the bacterial surface when strain QH-2 was incubated with 10 mM Cr(VI) after 18 h (Figure 2(b)).

### 3.2. Reducing Cr(VI) by Strain QH-2

**Figure 3:**
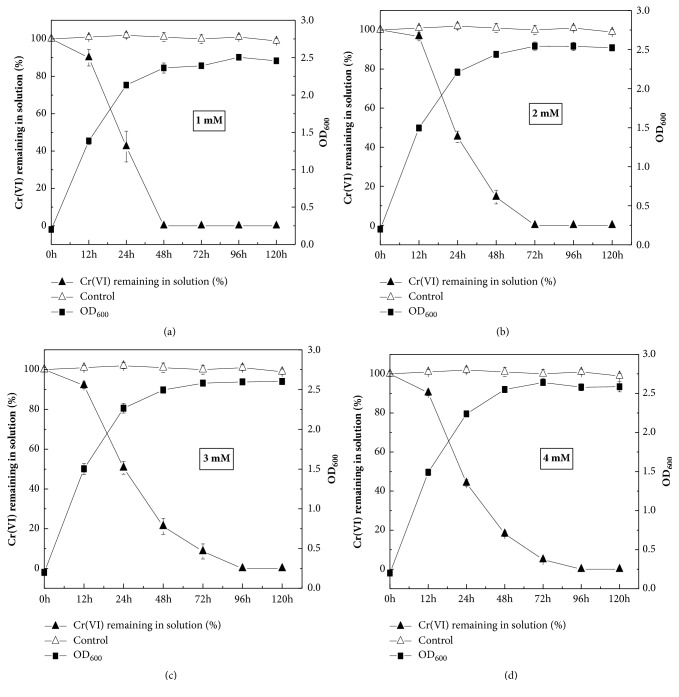
The capability of Cr(VI) reduction by strain QH-2 at 1mM, 2mM, 3mM, and 4mM of Cr(VI).

Selected pH of 9.0 used in this study was the optimum pH to grow and chosen according to the growth curves performed at different pH (Supplementary Data S1). The results of reduction Cr(VI) by strain QH-2 are exhibited in Figure 3. Strain QH-2 grew well, while the concentration of Cr(VI) was gradually reduced over time. The growth curves had no remarkable differences under 1 - 4 mM Cr(VI). Furthermore, complete Cr(VI) reductions were observed within 48 h under the treatments of 1 mM Cr(VI), within 72 h under the treatments of 2 mM Cr(VI), and within 96 h under the treatments of 3 mM Cr(VI).  Figure 3(d) showed that 4 mM Cr(VI) was almost 100% reduced when the media was incubated after 96 h. The reduction rate of strain QH-2 could reach 2.17 mg/L Cr(VI) h^−1^ under 4 mM Cr(VI) in media.

### 3.3. Reducing Cr(VI) by Strain QH-2 under Different pH

**Figure 4:**
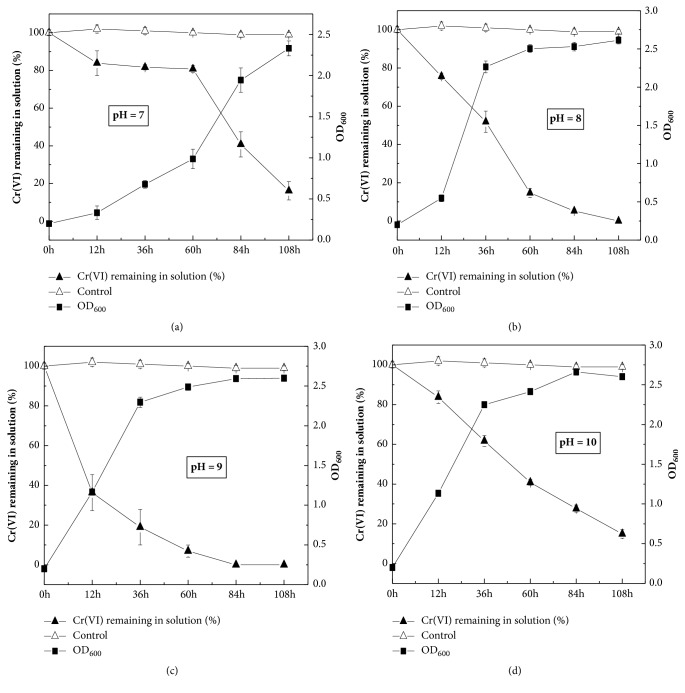
Effect of pH on the reduction of Cr(VI) at 5mM by strain QH-2.

The influences of pH (6 to 10) on the reducing Cr(VI) via strain QH-2 are showed in Figure 4. There were relative differences under different pH. When the pH of media was 6, strain QH-2 almost could not grow and reduce Cr(VI). In contrast, strain QH-2 could grow well at pH 7, 8, 9, and 10. The growth rates of strain QH-2 at pH 8, 9, and 10 were faster than that at pH 7 with 5 mM Cr(VI) (Figure 4). Results indicated that alkaline conditions were more favourable to reduce Cr(VI) by strain QH-2 than acidic and neutral conditions.  Figure 4(c) showed that the optimum pH to reduce Cr(VI) by strain QH-2 was 9. Furthermore, Cr(VI) could be completely reduced within 84 h. In contrast, the remaining Cr(VI) was 40.75% at pH 7, 5.21% at pH 8, and 27.66% at pH 10 at the same time point.

### 3.4. The Resistance of Strain QH-2 to Cr(VI)

**Figure 5:**
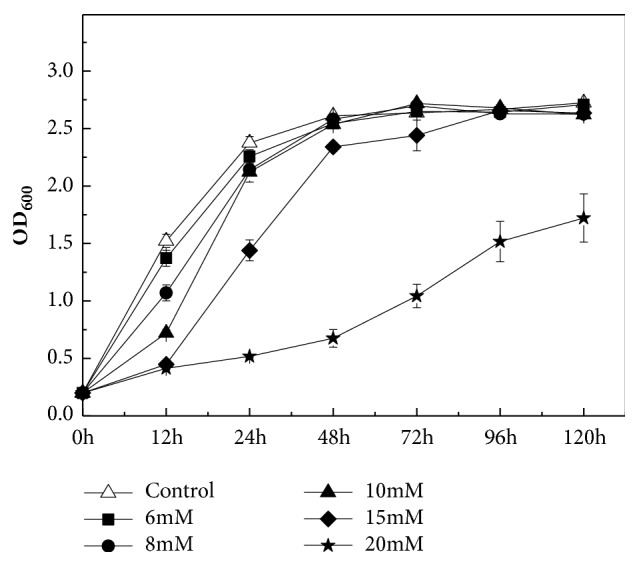
Evaluation of the resistance of strain QH-2 to 6mM, 8 mM, 10 mM, 15 mM, and 20mM Cr(VI).

As shown in Figure 5, strain QH-2 grew well under the treatment of 6 mM Cr(VI), compared with other treatments of Cr(VI). Furthermore, the growth of strain QH-2 was hardly affected up to 8 mM and 10 mM Cr(VI), except for a slight inhibition at the first 24 h of incubation. When the concentrations of Cr(VI) in media reached 15 mM, the inhibitory effect became obvious. The growth of strain QH-2 at 20 mM Cr(VI) was significantly inhibited during the entire incubation time when compared with the control. The results also showed that strain QH-2 could withstand the toxicity of Cr(VI) at certain concentrations.

## 4. Discussion

*Microbacterium* sp. QH-2 belongs to a new Cr(VI)-reducing bacterium that was isolated from the alkali soil contaminated with high concentration of Cr(VI). The soil is used for stacking Cr slag all the year round. This soil environment also determines that strain QH-2 has high resistance to Cr(VI). When strain QH-2 was incubated in the media supplemented with 10 mM Cr(VI) (Figure 2), the protrusions were observed on the bacterial surface. These changes might be caused by Cr(III) precipitates being attached to or adsorbed in the outer bacterial cells surface (Zhu et al., 2007) [38].

Members of the genus* Microbacterium* have been reported to have the capacity to reduce Cr(VI). For instance,* Microbacterium* MP30 could reduce 0.1 mM Cr(VI) less than 30 h with a reduction rate of 0.17 mg/L Cr(VI) h^−1^ [39]. When 50% tryptic soy broth was added with 2 mM Cr(VI),* Microbacterium* Cr-K29 and* Microbacterium* Cr-K20 had reduction rates of 2.17 and 0.52 mg/L Cr(VI) h^−1^, respectively [40].* Microbacterium* culture (X7) could reduce 100 mg/L Cr(VI) when media were under the incubation after 75 h (1.33 mg/L Cr(VI) h^−1^) [41]. According to the present study, strain QH-2 could reduce 68% of 10 mM Cr(VI) after 120 h in media (Supplementary Data S2). The capacity to reduce Cr(VI) of strain QH-2 is stronger than that of the above* Microbacterium* species.

Compared with the reduction rates of some species of Cr(VI)-reducing bacterial strains such as* Pseudochrobactrum asaccharolyticum *(KC618329) [30],* Bacillus *sp. MDS05 (EU236673) [32], and* Microbacterium *sp. (JN674183) [33] (Table 1), the reduction rate of strain QH-2 was significantly higher. Notably, the reduction rate was at a similar level with that of* Bacillus amyloliquefaciens* (JQ429762). Das et al. [12] reported that* Bacillus amyloliquefaciens* (JQ429762) could reduce only 56.5% of nearly 4 mM Cr(VI) after 144 h. By contrast, strain QH-2 could almost completely reduce 4 mM Cr(VI) after 96 h (Figure 3). These results indicated that strain QH-2 could reduce Cr(VI) more efficiently.

The pH value is an important factor to have influence on reducing Cr(VI). When the media were incubated after 108 h, 5 mM Cr(VI) was almost 100% reduced in the media at the initial pH of 8 and 9, while residual Cr(VI) was still existing at the initial pH of 7 and 10 (Figure 4). Sayel et al. [35] studied that Cr(VI) reduction under pH 8 and pH 10 was better than that under pH 7 and pH 11. However, the amount of Cr(VI) reduced by* Bacillus *sp. (FJ178872.) enhanced with the increase in the pH from 6 to 9 [42]. These differences might have been relevant with the different characteristics of Cr(VI)-reducing bacteria.

In this study, the optimum pH to reduce Cr(VI) for strain QH-2 is 9. Similarly, all the Cr(VI) was reduced by* Leucobacter *sp. CRB1 under pH 9 [1]. However,* Bacillus amyloliquefaciens* showed the best effect of reducing Cr(VI) at pH 7 [12]. Cheng and Li [32] found that the optimum pH to reduce Cr(VI) of* Bacillus *sp. was 8. In addition,* Enterococcus gallinarum* had the most obvious Cr(VI) reduction effect at pH 10 [35]. Furthermore, the maximum level to reduce Cr(VI) was shown at pH 10 by* Bacillus* sp. strain KSUCr5 [43]. The effect of reducing Cr(VI) by bacterial strains is obviously affected by pH [1]. In summary, the differences in optimum pH for Cr(VI) reduction by different bacterial strains might depend on the properties of the soil or effluent where the bacterial strains are isolated from.

Strain QH-2 also exhibited a relatively high tolerance even to 20 mM Cr(VI) (Figure 5). The resistance of strain QH-2 to Cr(VI) might be directly connected to long-term exposure to Cr(VI). The existence of strain QH-2 in Cr(VI)-contaminated alkali soil is also the result of natural selection. The mechanisms of Cr(VI) resistance included offsetting Cr^6+^-induced oxidative stress by activating reactive oxygen species scavenging enzymes and DNA repair, etc. [44, 45]. However, the resistance mechanism of strain QH-2 to Cr(VI) is still not explicit and a further study is needed.

As shown in Table 1, previous studies have reported the resistance to Cr(VI) of different bacterial strains. Soni et al. [33] reported that* Microbacterium *sp. (JN674183) was isolated from the medium supplemented with 19.2 mM Cr(VI). This meant that* Microbacterium *sp. (JN674183) could resist 19.2 mM Cr(VI). When compared with other strains, strain QH-2 exhibited a higher tolerance to Cr(VI), even at 20 mM. Although* Bacillus *sp. MDS05 (EU236673) could resist 48 mM Cr(VI) [32], its capacity to reduce Cr(VI) was weaker than that of strain QH-2. Furthermore, the reduction rate of* Bacillus *sp. MDS05 (EU236673) was lower than that of strain QH-2. These results indicate that strain QH-2 could exist in environments polluted with high concentrations of Cr(VI). Meanwhile, strain QH-2 has potential for the bioremediation of Cr(VI) in soil or effluent.

## 5. Conclusion

The bioremediation via Cr(VI)-reducing bacteria is a promising approach. The findings presented here indicate that* Microbacterium* sp. QH-2 has a fast reduction rate and relatively high tolerance to Cr(VI). These results mean that* Microbacterium* sp. QH-2 might have enough potential to detoxify Cr(VI), especially in Cr(VI)-contaminated alkali soil. Further studies will investigate the biological reduction mechanism and the resistance mechanism to Cr(VI) by* Microbacterium* sp. QH-2 and study the detoxification effects of* Microbacterium* sp. QH-2 in Cr(VI)-contaminated soil or effluent.

## Figures and Tables

**Table 1:** Comparisons of the capability of Cr(VI) reduction among the reported Cr(VI)-reducing bacterial strains.

Bacterial strains (the accession numbers in NCBI GenBank)	Initial Cr(VI) concentration (mM)	Reduction rate (%)	Reduction time	Reduction rate mg/L Cr(VI) h^−1^	Resistance to Cr(VI)	References
*Pseudochrobactrum asaccharolyticum *(KC618329)	1.92 mM	100	144 h	0.69	5 mM	[30]
*Stenotrophomonas maltophilia* ZA-6 (EU706282)	0.5 mM	100	56 h	0.46	16.5 mM	[31]
*Staphylococcus gallinarum* W-61 (EU706285)	0.25 mM	100	32 h	0.41	12.4 mM	[31]
*Bacillus *sp. MDS05 (EU236673)	0.19 mM	100	24 h	0.42	48 mM	[32]
*Microbacterium *sp. (JN674183)	0.2 mM	100	24 h	0.43	19.2 mM	[33]
*Intrasporangium* sp. Q5-1 (FJ487951)	0.98 mM	Nearly 100	24 h	1.99	17 mM	[34]
*Bacillus amyloliquefaciens* (JQ429762)	1.92 mM	100	45 h	2.22	17.3 mM	[12]
*Enterococcus gallinarum* (FR715561)	1.92 mM	100	48 h	2.08	9.6 mM	[35]
*Brucella *sp. (DQ437526)	0.96 mM	100	54 h	0.93	19.2 mM	[36]
*Microbacterium* sp. QH-2 (MH319855)	4 mM	100	96 h	2.17	20 mM	In this study

## Data Availability

All data included in this study are available upon request by contact with the corresponding author.

## Supplementary Materials

Supplementary Materials Supplementary figures provide additional information on the growth curves under different pH and the Cr(VI) reduction under different concentrations of Cr(VI). Specifically, Figure S1 shows the growth curves at different pH of strain QH-2. This can help to choose the optimum growth pH for the reducing experiments by strain QH-2. Figure S2 shows the Cr(VI) reduction of strain QH-2 at 6 mM, 7 mM, 8 mM, 10 mM, and 15 mM of Cr(VI). This can help to study the reducing capacity of strain QH-2 under high concentrations of Cr(VI).
