# Supplementary material for: Recognition of a New Cr(VI)-Reducing Strain and Study of the Potential Capacity for Reduction of Cr(VI) of the Strain
Source: Biomed Res Int. 2019 Feb 10;2019:5135017. doi: 10.1155/2019/5135017 (PMC6387719; doi:10.1155/2019/5135017)
Supplement: Supplementary Materials — Supplementary figures provide additional information on the growth curves under different pH and the Cr(VI) reduction under different concentrations of Cr(VI). Specifically, Figure S1 shows the growth curves at different pH of strain QH-2. This can help to choose the optimum growth pH for the reducing experiments by strain QH-2. Figure S2 shows the Cr(VI) reduction of strain QH-2 at 6 mM, 7 mM, 8 mM, 10 mM, and 15 mM of Cr(VI). This can help to study the reducing capacity of strain QH-2 under high concentrations of Cr(VI). [file 5135017.f1.docx]

**Recognition of a new Cr(VI)-reducing strain and study the potential capacity for reduction Cr(VI) of strain**

Chunyong Wang ^a, b^, Yanshan Cui ^a, b, ^[[1]](#footnote-1)^*^

^a^ College of Resources and Environment, University of Chinese Academy of Sciences, Beijing, 101408, People’s Republic of China

^b^ Research Center for Eco-environmental Sciences, Chinese Academy of Sciences, Beijing, 100085, People’s Republic of China

Fig.S1–The growth curves at different pH of strain QH-2.

Fig.S2–Cr(VI) reduction of strain QH-2 at 6 mM, 7 mM, 8 mM, 10 mM, and 15 mM of Cr(VI).

1. * Corresponding author at: College of Resources and Environment, University of Chinese Academy of Sciences, Huaibei Village 380, Huaibei Town, Huaibei District, Beijing 101408, China.

   E-mails: [cuiyanshan@ucas.ac.cn](mailto:cuiyanshan@ucas.ac.cn) (Yanshan Cui). Tel: +86-10- 88256467 [↑](#footnote-ref-1)
